# Patients’ Satisfaction With Medication Delivery Pharmacy Services in a Tertiary Hospital in Asir, Saudi Arabia: A Cross-Sectional Study

**DOI:** 10.7759/cureus.48903

**Published:** 2023-11-16

**Authors:** Amaal F Alshahrani, Ibrahim M Dighriri

**Affiliations:** 1 Department of Pharmacy, Armed Forces Hospital, Southern Region, Abha, SAU; 2 Department of Pharmacy, King Abdulaziz Specialist Hospital, Taif, SAU

**Keywords:** aseer, optimize healthcare, satisfaction level, medication delivery, pharmacy service

## Abstract

Background: Pharmaceutical care plays a crucial role in optimizing medication administration and improving patient health outcomes. However, medication adherence remains a challenge, with a significant percentage of patients discontinuing their medications. Value-added services (VASs), such as medication delivery, have been introduced to enhance pharmacy services and medication adherence.

Objective: This study aims to evaluate satisfaction with a new medication delivery service at an Armed Forces Hospital outpatient pharmacy in Saudi Arabia and identify factors impacting utilization.

Methods: A cross-sectional survey assessed patient satisfaction with a new pharmacy delivery service between January 2023 and March 2023. The target population consisted of adult patients who had used the pharmacy delivery service for at least one month. The survey contained 23 Likert scale questions assessing satisfaction across three domains: delivery process/personnel, medication quality, and pharmacist adherence to best practices.

Results: A total of 110 patients responded to the survey, 383 invited patients; the mean age was 51.2 ± 15.3, and most were male 92 (83.6%), married 97 (88.2%), and living in urban areas 63 (57.3%). The overall satisfaction rate was 97 (88.1%), with 67 (60.9%) reporting satisfaction with the medication delivery service. On the delivery process/personnel items, over half strongly agreed that the delivery person called before arriving 59 (53.6%), medications were received on time 58 (51.8%), and the delivery person was polite 64 (58.2%). Most strongly agreed that the service helped with adherence 70 (63.6%) and saved travel costs 72 (65.5%) for medication-quality items. Most also strongly agreed that medications were properly packaged 65 (59.1%) and labeled 71 (64.5%). Regarding pharmacist practices, approximately 56 (50.9%) strongly agreed that the pharmacist provided education materials, inquired about adherence 49 (44.5%), and was respectful 55 (50%). Bivariate analyses found no significant associations between satisfaction and age, gender, residence, education, marital status, income, or disease (all p > 0.05). Satisfaction remained uniformly high across subgroups.

Conclusion: The medication delivery service demonstrated excellent patient reception regardless of its characteristics. Overall satisfaction with these services was high. There was no association between sociodemographic characteristics and the level of satisfaction. Continued monitoring and refinement could maximize the quality of pharmaceutical care afforded through innovative models supporting medication adherence.

## Introduction

Pharmaceutical care is a critical discipline centered on optimizing the administration of medications to improve patient health outcomes [1]. These services offer immense potential benefits to patients, including improved communication, convenience, and ultimately increased utilization [2,3]. Enhanced medication adherence can lead to reduced hospitalization rates and lower mortality for those with chronic conditions [4,5]. However, it is concerning that approximately half of these patients discontinue their medications over time, with only 50-70% demonstrating high adherence [6,7], partly due to various patient, provider, and system-level factors [8,9]. In this context, patient-system interactions, especially prescription refills, play a pivotal role in medication adherence, with timely and successful refills being fundamental for optimal treatment adherence. Value-added services (VAS) were introduced to elevate the quality of prescription medication deliveries [10]. Patients now have the freedom to select their preferred VAS, such as integrated drug dispensing systems, appointment card services, mail pharmacy services, and drive-through pharmacy services. It is important to note that patient satisfaction significantly shapes the overall quality of healthcare services [11].

Studies have highlighted patients' dissatisfaction with the time it takes to receive their medications [12]. Extended waiting periods can significantly influence patients' perceptions of pharmacy service quality, subsequently impacting the success of their treatment [13]. Recent research by Almaghaslah et al. evaluating patient satisfaction with e-prescription services in Saudi Arabia shows that patients are favorably skewed towards pharmacy staff's knowledge, skills, approachability, and friendliness [14]. Similarly, Alfahad et al. found that customers using VAS reported substantially higher overall satisfaction scores compared to those using traditional services [15]. Furthermore, a study by Govender and Suleman discovered no significant differences in patient satisfaction between postal and community pharmacies in South Africa, though community pharmacies were often more effective in addressing specific patient concerns [16].

However, despite the growing body of evidence supporting the importance of pharmaceutical care and medication adherence [1,4], adherence remains a challenge, with a significant percentage of patients discontinuing long-term medications [6,7]. To address this issue, value-added services like prescription delivery have been introduced to enhance pharmacy services and medication adherence [10]. Existing research indicates that the utilization of such services can lead to increased patient satisfaction [17]. However, the satisfaction levels of patients using the recently introduced medication delivery service at Armed Forces Hospital in the Southern Region of Saudi Arabia have not been assessed. Since patient satisfaction is a crucial indicator of service quality and a predictor of medication adherence, this study aims to evaluate patient satisfaction with this new service and identify factors associated with satisfaction, as well as any potential barriers to service utilization. The evaluation of this new service delivery model holds significance in understanding its impact on the quality of pharmaceutical care provided to patients at Armed Forces Hospital.

## Materials and methods

Study design

This was a quantitative, cross-sectional study using a survey conducted in the Armed Forces Hospital Southern Region in Khamis Mushait, Saudi Arabia. It is a major hospital located in the city of Abha in southwestern Saudi Arabia. Abha is the capital of Aseer Province.

Setting and time period

The study was conducted in the outpatient pharmacy department of the Armed Forces Hospital Southern Region in Khamis Mushait. This tertiary care hospital provides comprehensive medical services to military personnel, their families, and civilians in the Asir region. The study was carried out over a three-month period, from January 2023 to March 2023.

Target population

The target population included all adult patients receiving outpatient medications through the new pharmacy delivery service at the study hospital. This service allows patients to receive periodic refills of non-acute medications delivered to their homes, reducing the need to visit the pharmacy in person.

Eligibility criteria

The inclusion criteria for participants were adult patients aged 18 years or older who had been recipients of the pharmacy home medication delivery service for at least one month and had the ability to complete an online survey in either Arabic or English. Patients were excluded if they were receiving short-term or acute medications not eligible for home delivery, were inpatients, or were emergency department patients.

Sample size

The sample size was determined using the Raosoft sample size calculator (Raosoft®, Raosoft, Inc., Windermere Road, Seattle, WA, available at: http://www.raosoft.com/samplesize.html), with a 5% margin of error, 95% confidence level, 50% response distribution, and a population size of approximately 5,000 based on pharmacy data. This yielded the minimum recommended sample size of 383 patients.

Recruitment and data collection

Patients meeting the inclusion criteria were identified from pharmacy records and invited to participate via social media messages containing a link to the online survey. Three reminders were sent to non-respondents at two-week intervals.

Survey development

Based on a review of existing pharmacy satisfaction questionnaires, the researchers developed the survey instrument. It was piloted with 10 patients and refined prior to broader distribution. The final survey was anonymous and voluntary, available in both Arabic and English. The survey consists of two sections. The first section is concerned with gathering information about sociodemographic characteristics. The second section of the questionnaire consisted of 23 statements with responses in the form of a 5-item Likert scale and was concerned with assessing the level of the participants’ satisfaction regarding the quality of the service. This section was further subdivided into three sections concerned with assessing the participants’ satisfaction with the delivery process and delivery personnel (4 statements), the quality of the delivered medications (8 statements), and the pharmacist's adherence to best practices (11 statements). Therefore, the total score a participant can achieve ranges from 0 to 92 points. The level of satisfaction was categorized into five categories: very dissatisfied (0-19 points), dissatisfied (20-39 points), somewhat satisfied (40-59 points), satisfied (60-79 points), or very satisfied (80-92 points).

Data analysis

All analyses were performed using the Statistical Package for the Social Sciences (SPSS version 26, IBM Corp., Armonk, NY). Descriptive statistics such as frequencies, percentages, means, and standard deviations were calculated to summarize the sample characteristics and responses to the survey questions. Participant demographic and clinical characteristics were summarized using frequencies and percentages for categorical variables. Means and standard deviations were calculated for continuous variables such as age. Overall satisfaction scores were calculated by summing the participant's responses to the Likert scale items, with possible total scores ranging from 0 to 92. Frequencies and percentages were used to categorize participants' overall level of satisfaction according to the predetermined categories (very dissatisfied, somewhat satisfied, satisfied, and very satisfied). To examine factors associated with satisfaction, participants were classified as either "dissatisfied" or "satisfied" based on their total satisfaction score. Bivariate analyses using chi-square tests were conducted to explore potential associations between demographic and clinical characteristics and satisfaction levels. Exact tests were used when expected cell counts were less than 5. Statistical significance was set at p < 0.05. The results of the statistical analyses will be displayed in tables and figures as appropriate to the data.

Ethical considerations

The study protocol received ethical approval from the King Khalid University Bioethics Committee under reference number ECM#2022-3116 and approval from the research ethics committee in the Armed Forces Hospital Southern Region. Participants were provided with an online information letter explaining the study and seeking their voluntary, informed consent. This introductory cover page preceded the survey and stated that involvement was optional, responses were confidential, and participants could withdraw at any time without giving a reason.

## Results

Out of 383 patients who were invited, 110 of them responded to the survey and completed it. Participants' ages ranged from 18 to more than 45 years, with a mean age of 51.2 ± 15.3 years. Exactly 92 (83.6%) were males, and 63 (57.3%) lived in urban areas. As for educational level, 40 (36.4%) had a below-secondary level of education, while 40 (36.4%) had a university level of education. A total of 97 (88.2%) were married, 37 (33.6%) had a monthly income exceeding 8000 Saudi riyals (SR) (1 USD = 3.75 SR), and 24 (21.8%) had a monthly income of less than 3000 SR. As for diseases for which medication is dispensed, the most reported were cardiovascular diseases 51 (46.4%), followed by bone and joint diseases 26 (23.6%), gastrointestinal tract (GIT) diseases 19 (17.3%), eye diseases 17 (15.5%), and diseases of the kidneys and urinary system 14 (12.7%) (Table 1).

**Table 1:** Sociodemographic data of study participants (N = 110). N: number, (%): percentage, SR: Saudi Riyal; CVD: cardiovascular disease; GIT: gastrointestinal tract.

Sociodemographic data	N	%
Age in years		
18–25	4	3.6%
26–35	17	15.5%
36–45	29	26.4%
>45	60	54.5%
Gender		
Male	92	83.6%
Female	18	16.4%
Residence		
Urban	63	57.3%
Rural	47	42.7%
Educational level		
Below secondary	40	36.4%
Secondary	30	27.3%
University	40	36.4%
Marital status		
Single	5	4.5%
Married	97	88.2%
Divorced widow	8	7.3%
Monthly income		
<3000 SR	24	21.8%
3000–8000 SR	49	44.5%
>8000 SR	37	33.6%
Diseases for which medication is dispensed		
CVD	51	46.4%
Bone and joint diseases	26	23.6%
GIT diseases	19	17.3%
Eye diseases	17	15.5%
Diseases of the kidneys and urinary system	14	12.7%
Respiratory diseases	13	11.8%
Skin diseases	8	7.3%
Tumors and cancers	4	3.6%
Others	48	43.6%

Table 2 demonstrates the questions related to satisfaction with medication delivery pharmacy services among patients. Regarding the delivery process and delivery personnel, more than half of the participants, 59 (53.6%), strongly agreed that the delivery person called them before delivering the medicines to find out the right time for the participant. Similarly, slightly more than half of the participants, 57 (51.8%), strongly agreed that they had received the medicines in a timely manner. Furthermore, more than half of the participants, 64 (58.2%), strongly agreed that the delivery person handed them the medicines in a polite manner. Finally, about two-thirds of the participants, 67 (60.9%), strongly agreed that the delivery employee was polite and respectful.

In terms of the quality of the delivered medications, about two-thirds of the participants, 72 (65.5%) strongly agree that the medication delivery service helped them save on travel expenses to and from the hospital. Nearly the same proportion of the participants, 70 (63.6%) strongly agreed that the medication delivery service helped them take their medications regularly. About one-half of the participants, 57 (51.8%), strongly agreed that they got all the medications that were prescribed to them. Similarly, more than one-half of the participants, 65 (59.1%), strongly agreed that all the medicines they received were well packaged. In the same context, about two-thirds of the participants, 68 (61.8%), strongly agreed that all the medicines they received were fine.

Furthermore, about two-thirds of the participants, 71 (64.5%), strongly agreed that a label explaining the instructions for each medication was attached. Similarly, about two-thirds of the participants, 66 (60.0%), strongly agreed that the instructions describing the patient's name, the name of the medication, and how to use it were clearly written. Finally, slightly more than one-half of the participants, 58 (52.7%), strongly agreed that they have received a copy of the medical prescription containing all the medicines prescribed to them, which they can use to aid in acquiring medicines from external pharmacies.

Regarding the pharmacists’ adherence to the best practices, slightly less than one-half of the participants, 50 (45.5%) strongly agreed that the pharmacist contacted them before sending the medicine. About one-half of the participants, 56 (50.9%), strongly agreed that the pharmacist provided them with written or printed information about the treatment and about the disease for which the medication was prescribed. More than one-third of the participants, 45 (40.9%), strongly agreed that the pharmacist called them to make sure that the medicines were delivered to them properly. Nearly the same proportion of the participants (40.0%) strongly agreed that the pharmacist showed them how to know if the medicines were effective.

More than one-third of the participants, 47 (42.7%), strongly agreed that the pharmacist explained to them all possible side effects. The same proportion of participants, 47 (42.7%) strongly agreed that when the pharmacist evaluated their treatment, he or she considered their medical history and the medications they had previously used. Slightly less than one-half of the participants, 49 (44.5%) strongly agreed that the pharmacist inquired about their commitment to take the prescribed medications according to the attached instructions. More than one-third of the participants, 42 (38.2%), strongly agreed that the pharmacist provided them with the necessary information on the proper storage of medicines. Nearly the same proportion of the participants, 41 (37.3%), strongly agreed that the pharmacist provided them with comprehensive medication counseling and encouraged them to ask any relevant questions. Slightly less than one-half of the participants, 48 (43.6%) strongly agreed that the pharmacist talked to them in plain language, and they were able to understand everything he or she had to say. Finally, almost one-half of the participants, 55 (50.0%), strongly agreed that the pharmacist was courteous and respectful.

**Table 2:** Satisfaction toward medication delivery pharmacy services among patients. N: number; (%): percentage.

Patients' satisfaction	Strongly disagree		Disagree		Neutral		Agree		Strongly agree	
	No	%	N	%	N	%	N	%	N	%
Delivery process and delivery personnel										
The delivery person called me before delivering the medicines to find out the right time for me	7	6.4%	11	10.0%	7	6.4%	26	23.6%	59	53.6%
I received the medicines in a timely manner	12	10.9%	9	8.2%	7	6.4%	25	22.7%	57	51.8%
The delivery person handed me the medicines in a polite manner	9	8.2%	4	3.6%	5	4.5%	28	25.5%	64	58.2%
The delivery employee was polite and respectful	8	7.3%	4	3.6%	7	6.4%	24	21.8%	67	60.9%
The quality of the delivered medications										
Medication delivery service helped me save on travel expenses to and from the hospital	6	5.5%	6	5.5%	2	1.8%	24	21.8%	72	65.5%
Medication delivery service helped me to take my medications regularly	6	5.5%	7	6.4%	5	4.5%	22	20.0%	70	63.6%
I got all the medication that was prescribed to me	11	10.0%	9	8.2%	6	5.5%	27	24.5%	57	51.8%
All the medicines I received were well packaged	7	6.4%	2	1.8%	8	7.3%	28	25.5%	65	59.1%
All the medications I received were fine	6	5.5%	1	.9%	4	3.6%	31	28.2%	68	61.8%
Labels explaining the instructions for each medication have been placed	4	3.6%	0	0.0%	2	1.8%	33	30.0%	71	64.5%
The instructions describing the patient's name, the name of the medication, and how to use it are clearly written	5	4.5%	3	2.7%	7	6.4%	29	26.4%	66	60.0%
I received a copy of the medical prescription containing all the medicines prescribed to me, which I can use to dispense medicines from external pharmacies	9	8.2%	11	10.0%	5	4.5%	27	24.5%	58	52.7%
The pharmacist adherence to the best practices										
The pharmacist contacted me before sending the medicine	11	10.0%	7	6.4%	8	7.3%	34	30.9%	50	45.5%
The pharmacist provided me with written/printed information about the treatment and the diseases I suffer from	12	10.9%	10	9.1%	7	6.4%	25	22.7%	56	50.9%
The pharmacist called me to make sure that the medicines were delivered to me properly	17	15.5%	17	15.5%	9	8.2%	22	20.0%	45	40.9%
The pharmacist showed me how to know if the medicines were effective	16	14.5%	18	16.4%	7	6.4%	25	22.7%	44	40.0%
The pharmacist explained to me all possible side effects	20	18.2%	16	14.5%	6	5.5%	21	19.1%	47	42.7%
When the pharmacist evaluates my treatment, he considers my medical history and the medications I've used previously	14	12.7%	15	13.6%	8	7.3%	26	23.6%	47	42.7%
The pharmacist inquired about my commitment to take the prescribed medications according to the attached instructions	14	12.7%	15	13.6%	8	7.3%	24	21.8%	49	44.5%
The pharmacist provided me with the necessary information on the proper storage of medicines	17	15.5%	11	10.0%	10	9.1%	30	27.3%	42	38.2%
The pharmacist provided me with comprehensive medication counseling and encouraged me to ask any relevant questions	17	15.5%	14	12.7%	11	10.0%	27	24.5%	41	37.3%
The pharmacist talked in a plain language, and I could understand everything he had to say	12	10.9%	8	7.3%	8	7.3%	34	30.9%	48	43.6%
The pharmacist was courteous and respectful	10	9.1%	5	4.5%	8	7.3%	32	29.1%	55	50.0%

**Figure 1:**
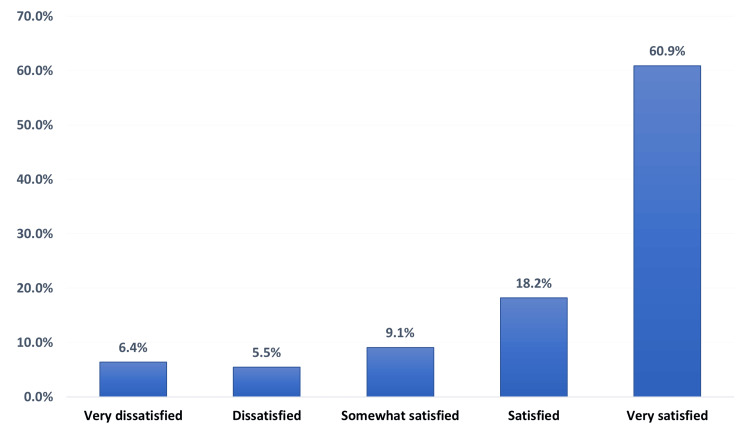
Overall satisfaction toward medication delivery pharmacy services among patients.

Figure 1 illustrates the overall satisfaction with medication delivery pharmacy services among patients. The overall satisfaction rate among the participants was 97 (88.1%). About two-thirds of the participants, 67 (60.9%), were very satisfied regarding medication delivery service, whereas less than one-fifth, 20 (18.2%), were satisfied, 10 (9.1%) were somewhat satisfied, and a minority of the participants, 13 (11.9%), were either dissatisfied or strongly dissatisfied, 6 (5.5%) and 7 (6.4%), respectively, regarding their medication delivery service.

Factors associated with satisfaction regarding medication delivery service are listed in Table 3. The age of patients showed no significant association with satisfaction levels (p = 0.717). In the 18-25-year-old age group, all four patients (100%) reported satisfaction. Among the 26-35-year-old age group, 16 out of 17 (94.1%) were satisfied. In the 36-45-year-old age group, 25 out of 29 (86.2%) expressed satisfaction. Finally, among those aged over 45 years, 52 out of 60 patients (86.7%) were satisfied with their medication delivery. Gender did not significantly influence satisfaction (p = 0.486). Out of 92 male patients, 82 (89.1%) reported satisfaction, while 15 out of 18 female patients (83.3%) were satisfied. Residence was not a significant factor in patient satisfaction (p = 0.790). Of the 63 patients living in urban areas, 56 (88.9%) were satisfied, while in rural areas, 41 out of 47 patients (87.2%) reported satisfaction. Educational level did not significantly affect satisfaction levels (p = 0.937). Of the patients with lower secondary education, 35 out of 40 (87.5%) reported satisfaction. Among those with secondary education, 27 out of 30 (90.0%) were satisfied. And 35 out of 40 patients (87.5%) with university-level education were satisfied.

Marital status did not significantly influence satisfaction (p = 0.363). All 5 single patients (100%) reported satisfaction, while 86 out of 97 married patients (88.7%) were satisfied. Among divorced or widowed patients, 6 out of 8 (75.0%) reported satisfaction. Monthly income did not significantly affect satisfaction levels (p = 0.889). Of the patients earning less than 3000 SR, 21 out of 24 (87.5%) were satisfied. Among those earning between 3000 and 8000 SR, 44 out of 49 (89.8%) reported satisfaction. For those earning more than 8,000 SR, 32 out of 37 (86.5%) were satisfied. The type of disease for which the medication was dispensed did not significantly influence satisfaction levels (p = 0.423). Among patients with cardiovascular diseases, 41 out of 51 (80.4%) reported satisfaction. For bone and joint diseases, 23 out of 26 (88.5%) were satisfied. All eight patients with skin diseases and all four patients with tumors or cancers (100%) reported satisfaction. Of the patients with eye diseases, 14 out of 17 (82.4%) were satisfied. For diseases of the kidneys and urinary system, 12 out of 14 (85.7%) reported satisfaction. Among patients with gastrointestinal diseases, 16 out of 19 (84.2%) were satisfied. For respiratory diseases, 11 out of 13 (84.6%) reported satisfaction. Finally, 41 out of 48 patients (85.4%) with other diseases reported satisfaction. In conclusion, none of the factors examined in this study showed a significant association with patient satisfaction regarding medication delivery. However, high satisfaction rates were reported across all groups, indicating that the medication delivery system is generally well-received among patients.

**Table 3:** Factors associated with patients' satisfaction regarding medication delivery. N: number; (%): percentage; SR: Saudi Riyal; CVD: cardiovascular disease; GIT: gastrointestinal tract; P: Pearson X^2^ test; $: exact probability test.

Factors	Satisfaction				P-value
	Dissatisfied		Satisfied		
	No	%	No	%	
Age in years					0.717^$^
18-25	0	0.0%	4	100.0%	
26-35	1	5.9%	16	94.1%	
36-45	4	13.8%	25	86.2%	
>45	8	13.3%	52	86.7%	
Gender					0.486
Male	10	10.9%	82	89.1%	
Female	3	16.7%	15	83.3%	
Residence					0.790
Urban	7	11.1%	56	88.9%	
Rural	6	12.8%	41	87.2%	
Educational level					0.937^$^
Below secondary	5	12.5%	35	87.5%	
Secondary	3	10.0%	27	90.0%	
University	5	12.5%	35	87.5%	
Marital status					0.363^$^
Single	0	0.0%	5	100.0%	
Married	11	11.3%	86	88.7%	
Divorced/widow	2	25.0%	6	75.0%	
Monthly income					0.889^$^
<3000 SR	3	12.5%	21	87.5%	
3000-8000 SR	5	10.2%	44	89.8%	
>8000 SR	5	13.5%	32	86.5%	
Diseases for which medication is dispensed					0.423^$^
CVD	10	19.6%	41	80.4%	
Bone and joint diseases	3	11.5%	23	88.5%	
Skin diseases	0	0.0%	8	100.0%	
Tumors and cancers	0	0.0%	4	100.0%	
Eye diseases	3	17.6%	14	82.4%	
Diseases of the kidneys and urinary system	2	14.3%	12	85.7%	
GIT diseases	3	15.8%	16	84.2%	
Respiratory diseases	2	15.4%	11	84.6%	
Others	7	14.6%	41	85.4%	

## Discussion

Medication delivery services play a pivotal role in the healthcare system by ensuring timely and appropriate access to medications, which can subsequently influence patient adherence to therapeutic regimens [18,19]. This study provides valuable insights into patients' satisfaction with medication delivery pharmacy services. The results of this study revealed that around two-thirds of the participants (60.9%) were very satisfied with the medication delivery provided. On the other hand, only 6.5% of participants were very dissatisfied with the service. These findings are consistent with the findings reported by Ali et al., who evaluated patient satisfaction with ambulatory care pharmacy services at two tertiary care facilities and reported that 48.2% of the participants were very satisfied, whereas very few respondents (10.2%) were dissatisfied with the pharmacy services [20].

Most participants showed strong agreement that they were satisfied with the delivery personnel's timeliness and politeness. The quality of the delivered medications, as indicated by the packaging and the state of the medications, received positive feedback from the majority. Crucially, over 60% believed the service aided in ensuring they took their medications regularly and saved on travel expenses. This not only shows the efficiency of such services but also suggests potential economic benefits for the patients, especially when considering the potential costs of missed medications and subsequent hospital visits. Respondents scored very well in the level of satisfaction with adherence to the best practices (40.0-45.5% strongly agreed). This was consistent with the findings reported by Ali et al. [20]. In contrast, a recent study in the Al-Jouf province of northern Saudi Arabia reported lower scores in medication counseling [21]. Satisfaction with the pharmacist's adherence to best practices, although generally positive, was slightly less pronounced compared to delivery-related aspects. The pharmacist's role in ensuring proper use, understanding, and adherence to medications is of paramount importance [22]. The findings suggest that while many patients are satisfied with the information and guidance they receive, there's still room for improvement, especially in areas like explaining potential side effects and the efficacy of medications.

The majority (88.1%) were either very satisfied or satisfied with the medication delivery service. Such high levels of satisfaction indicate that medication delivery services are well-received and potentially crucial in improving medication adherence, especially among patients who might face difficulties physically visiting the pharmacy [23]. Furthermore, the majority felt that the service helped them save on travel expenses and ensure a regular supply of medication(s). Both of these factors can indirectly contribute to better medication adherence [24]. Previous studies have suggested that ease of access to medications, reduced costs, and timely delivery can indeed improve adherence [24,25]. Also, satisfaction with labeling and packaging reflects the commitment to patient safety and the provision of clear instructions, which are crucial for therapeutic success. However, there are areas that could benefit from further improvements. While a significant number of patients received adequate information from the pharmacist, a considerable proportion felt they could be better informed, especially concerning side effects, treatment evaluation, and medication counseling, ensuring that patient education is vital to maximize therapeutic outcomes and minimize adverse reactions. The current study revealed an insignificant association between sociodemographic factors and the level of satisfaction (p ≥ 0.05 for all variables). Conversely, our finding was inconsistent with the findings conducted by Salamatullah et al., who reported that higher satisfaction levels were associated with high school education (p = 0.016) [5].

However, this study is not without its limitations. One limitation of this study is the relatively small sample size of 110 patients from a single hospital pharmacy department. The study relied on patient self-reports of medication adherence and satisfaction levels, which can be subject to recall bias and social desirability bias. The cross-sectional design only provides a snapshot of satisfaction at one point in time, without accounting for changes over the course of utilizing the pharmacy delivery service. Finally, the study was limited to adult patients at a military hospital in Saudi Arabia, so the results may not be generalized to other patient populations in different healthcare settings or geographic regions. Despite these limitations, the study provides initial evidence regarding satisfaction with a new medication delivery service model in an understudied population. Medication delivery services offer considerable value in the current healthcare landscape. With continuous feedback and regular improvements, such systems can revolutionize the way patients access their medications, leading to better adherence and improved health outcomes. It would be interesting to conduct further research on a larger scale and in different regions to understand if these findings hold universally.

## Conclusions

The study showed that the overall level of satisfaction of patients with medication delivery pharmacy services was high. Participants particularly appreciated the convenience of having medications delivered to their homes, which helped promote adherence, as well as the polite and helpful interactions with delivery personnel. No demographic or clinical characteristics significantly influenced satisfaction levels, suggesting the delivery model was well-received across different patient groups. While the findings provide initial support for medication delivery services as an option to enhance care quality and access, further research could explore satisfaction over longer time periods, assess clinical outcomes, and identify ways to continuously enhance a patient's experience. Overall, the high satisfaction demonstrated in this study indicates this innovative approach shows promise for supporting medication adherence through optimized pharmaceutical care.
